# The development of a provincial multidisciplinary framework of consensus-based standards for Point of Care Ultrasound at the University of Saskatchewan

**DOI:** 10.1186/s13089-019-0142-7

**Published:** 2019-10-17

**Authors:** Paul Olszynski, Daniel J. Kim, Irene W. Y. Ma, Michelle Clunie, Peggy Lambos, Tom Guzowski, Matthew Butz, Brent Thoma

**Affiliations:** 1 Department of Emergency Medicine, University of Saskatchewan, Saskatoon, Canada; 2 Department of Emergency Medicine, University of British Columbia, Vancouver, Canada; 3 Department of Medicine, University of Calgary, Calgary, Canada; 4 Department of Anesthesia and Perioperative Medicine, University of Saskatchewan, Saskatoon, Canada; 5 Department of Pediatrics, University of Saskatchewan, Prince Albert, Canada; 6 Department of Internal Medicine, University of Saskatchewan, Saskatoon, Canada; 7 Department of Family Medicine, University of Saskatchewan, Regina, Canada

**Keywords:** Point of Care Ultrasound, Framework, Standards, Multidisciplinary, Consensus, Quality assurance

## Abstract

**Objectives:**

The development and adoption of Point-of-Care Ultrasound (POCUS) across disciplines have created challenges and opportunities in implementing training and utilization standards. Within the context of a large, geographically disparate province, we sought to develop a multidisciplinary POCUS framework outlining consensus-based standards.

**Methods:**

A core working group of local POCUS leaders from Anesthesia, Emergency Medicine, Family Medicine, Intensive Care, Internal Medicine, Pediatrics, and Trauma, in collaboration with western Canadian colleagues, developed a list of key domains for the framework along with a range of potential standards for each area. The members of the working group and the registrants for a multidisciplinary Roundtable discussion at the University of Saskatchewan’s annual POCUS conference (SASKSONO19, Saskatoon, Saskatchewan, March 2nd, 2019) were invited to complete a survey on POCUS standards for each domain. The survey results were presented to and discussed by participants at the Roundtable discussion at SASKSONO19 who reached consensus on modified standards for each domain. The modified standards were considered for endorsement by all conference attendees using an audience-response system.

**Results:**

The working group proposed standards in eight domains: scope of use, credentialing and privileges, documentation, quality assurance, leadership and governance, teaching, research, and equipment maintenance. Consensus on modified standards was achieved in the 18 participant Roundtable. Each standard was then endorsed by > 90% of conference respondents.

**Conclusion:**

The resulting framework will inform the utilization of POCUS within Saskatchewan. Both this process and its outcomes could inform the development of multidisciplinary POCUS standards within other jurisdictions.

## Introduction

Point of Care Ultrasound (POCUS) is defined as “diagnostic or procedural guidance ultrasound that is performed by a clinician during a patient encounter to help guide the evaluation and management of that patient.” [1] It can impact a range of patient-related outcomes including diagnostic accuracy [2–6], time to diagnosis [7, 8], time to definitive management [8–10], procedural safety [11–16], decreased complications [15, 16], morbidity [17, 18], and mortality [19]. Several disciplines have developed guidelines and standards for the use of POCUS in clinical practice [12, 13, 20–25]. These POCUS standards offer guidance for evidence-based application, general training requirements, documentation standards, quality assurance, and equipment maintenance. As more healthcare disciplines adopt POCUS, it will be increasingly common for healthcare providers to exchange POCUS findings during transitions in care.

POCUS is used by a wide range of disciplines. While individual applications and specific uses of POCUS vary between disciplines, domains such as scope of use, training, and governance are similar and amenable to forming a common standard [26]. Multidisciplinary POCUS collaboration and consensus on standards may help ensure consistent patient care and support optimal POCUS training within our clinical context. The establishment of a common framework of standards for POCUS users may also increase the quality of POCUS scans and improve communication regarding patient findings. Given that some disciplines have more extensive experience in the integration of POCUS into clinical practice, we anticipate that the creation of a multidisciplinary, mutually agreed upon POCUS framework will facilitate the spread of best practices between disciplines.

We sought to develop and build consensus around a local multidisciplinary framework of consensus-based POCUS standards. We believe that this process could be utilized by other institutions and/or health associations to develop their own set of multidisciplinary POCUS standards.

## Methods

**Fig. 1:**
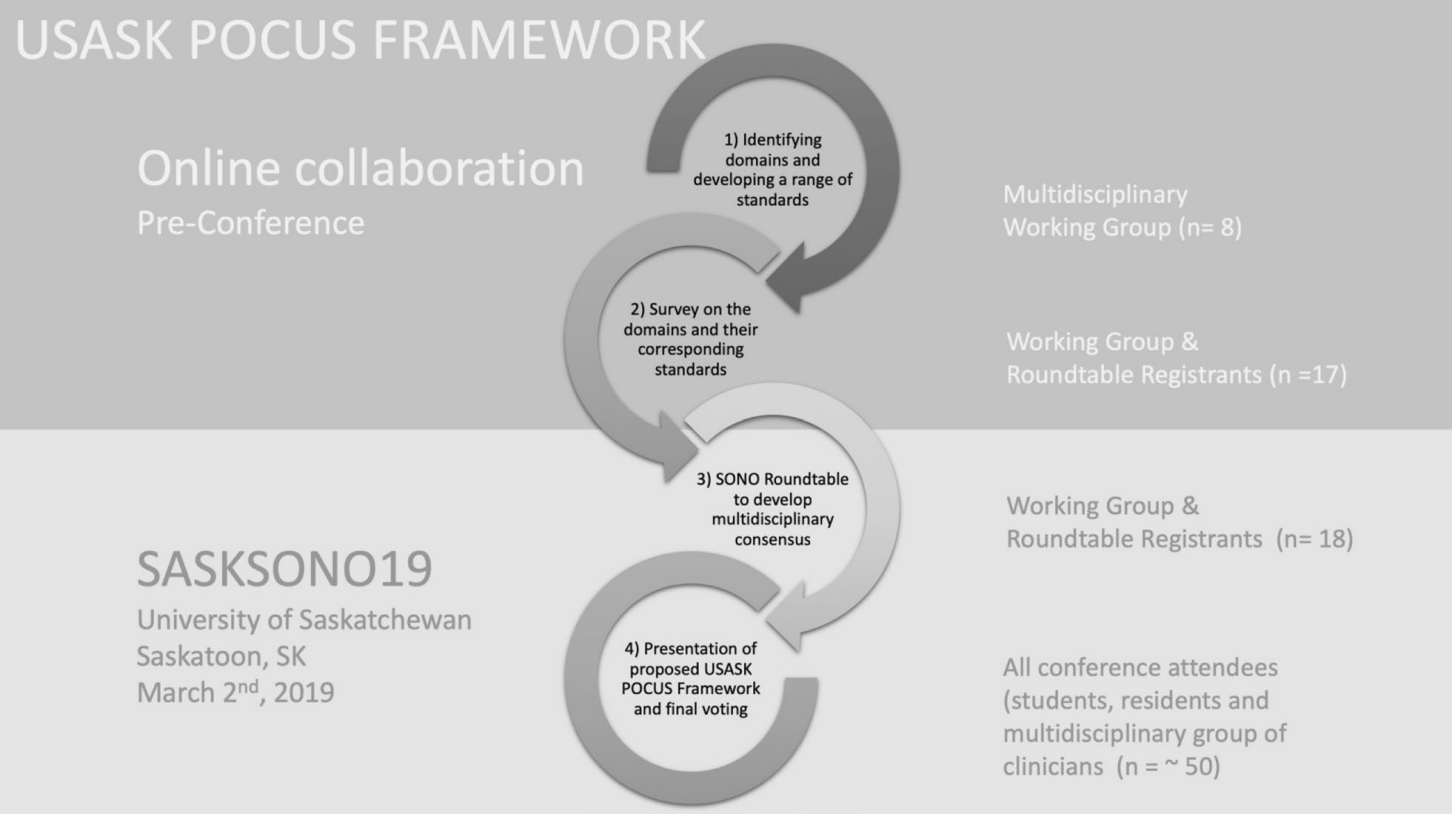
Four-part process to draft and build consensus around a multidisciplinary framework of POCUS standards

Collectively, we developed an iterative, four-part process to draft and build consensus around a multidisciplinary framework of POCUS standards (Fig. 1). Our process was informed in part by similar consensus-based processes carried out by colleagues in other fields [27, 28]. A Research Ethics Board exemption was sought and obtained from the University of Saskatchewan’s Research Ethics Board (BEH 957). Participation in any of the aspects of the framework process was voluntary and consent was implied through participation.

First, a working group of local POCUS leaders from Anesthesia, Critical Care, Emergency Medicine, Family Medicine, Internal Medicine, Pediatrics, and Trauma was identified based upon their leadership roles in the adoption of POCUS within their discipline in Saskatchewan and subsequently invited to participate via email invitations. The expertise within this group was buttressed through collaboration with western Canadian POCUS leaders that had been invited to present at the University of Saskatchewan’s annual POCUS Conference (SASKSONO19, Saskatoon, Saskatchewan, March 2nd, 2019). We reviewed other previously published domain-specific POCUS guidelines to develop a list of key domains for a multidisciplinary framework [12, 13, 20–25]. Once the domains had been determined, a range of potential standards for each domain was proposed by one working group member (PO). These potential standards were disseminated to the working group who reviewed and edited them until it was felt that they represented a reasonable possible standard that could be considered (Additional file 1: Appendix S1).

Second, members of the working group along with the registrants for a Roundtable discussion at SASKSONO19 were invited to complete a survey of the aforementioned proposed standards. Roundtable participants were required to be clinicians who use POCUS regularly in their practice. The survey was hosted on Google Forms (Google, Mountain View, CA) and asked each participant to indicate their preferred standard from the range of potential standards developed for each domain. ‘Other’ was also an option within each domain and, when selected, participants were invited to input alternative standards in free text.

Third, the results of this survey were presented to the participants who attended the Roundtable discussion at SASKSONO19. The results for each domain, including both the vote totals for the range of standards and the standards that were proposed within the survey were reviewed. Where there was a lack of clear consensus on one of the proposed standards, a detailed discussion occurred with the goal of drafting a standard for each domain that all the participants could support. Ultimately, consensus was achieved within the working group on a single standard for each domain.

Finally, the single modified standard supported by the members of the Roundtable discussion for each of the domains was reviewed by the conference attendees at the final plenary session of the conference. The rationale for each standard was explained, and conference attendees (including students, residents, and clinicians from multiple disciplines) indicated whether or not they supported each proposed standard using an audience-response system (Mentimeter, Stockholm, Sweden). We defined consensus a priori as an endorsement by > 80% of the respondents in each of our iterative consensus process (working group members, roundtable participants and conference attendees).

## Results

The eight working group participants developed a list of nine domains to be addressed in the framework (scope of use, credentialing and privileges, documentation, quality assurance, leadership and governance, teaching, research, and equipment maintenance) and proposed three potential standards for each (Additional file 1: Appendix S1).

The survey of the potential standards sent to the working group members and Roundtable registrants was completed by 17 clinicians (Table 1). Survey results (Table 2) revealed unanimous agreement in some domains (e.g., Scope of Use: The appropriate application of POCUS should be defined by individual disciplines and be used whenever supported by reasonable evidence) whereas there were a range of opinions and suggestions on other domains (e.g., Documentation standards). The standards that were suggested for each domain are outlined in Additional file 2: Appendix S2.

**Table 1:** Discipline of working group and roundtable participants

Participants	Working group (*n* = 8)		Roundtable (*n* = 18)	
Saskatchewan-based	EM IM Anesthesia Pediatrics	3 1 1 1	EM IM Anesthesia Critical care Pediatrics FM NP acute care	7 1 3 1 1 3 1
Invited from out-of-province	EM IM	1 1	EM	1

*EM* emergency medicine, *IM* internal medicine, *FM* family medicine, *NP* nurse practitioner

**Table 2:** Results of the pre-conference survey of the potential standards^a^ for each domain in order of preference

Domain	Preferred option^a^	Support (*n* = 17) (%)
Scope of use	1. The appropriate application of POCUS should be defined by individual specialties/disciplines and be used whenever supported by reasonable evidence	100
Credentials and privileges	1. Departments should define specific credentials that are required to receive privileges to use POCUS	35
	2. No credentials or additional privileges should be required for the use of POCUS; its use should be up to the clinician similar to any other aspect of the clinical assessment	25
	3. Any additional training required (and associated privileges) to use POCUS should be determined on a case-by-case basis by each department	18
Documentation	1. POCUS findings should be documented, and images captured when they play a significant role in patient care decisions	59
	2. POCUS findings should be documented, and all images should be captured for inclusion in the patient’s medical record	25
	3. POCUS should be documented in the same way as physical exam findings as part of the overall clinical assessment	18
Quality assurance	1. An audit of POCUS should be coordinated by any groups utilizing POCUS. Review of images, when available, is strongly encouraged	88
	2. POCUS use should include image capture and all images must be reviewed for quality assurance purposes. (12%)	12
Leadership and governance	1. Multidisciplinary committee with representatives from each specialty/discipline using POCUS	59
	2. Each specialty/discipline oversees its own use (41%)	41
Teaching	1. POCUS education can be provided by those with privileges recognized by their department	41
	2. POCUS education can be provided only by those with specific credentials as determined by a multidisciplinary POCUS committee	35
	3. POCUS education can be provided by clinicians without specific credentials	6
Research	1. Concerted and coordinated efforts to maximize research productivity to help propel USASK as a leader in POCUS research	59
	2. Clinical and educational/training research should be encouraged within each department	41
Equipment support and maintenance	1. Universal standards for POCUS equipment support and maintenance should be coordinated centrally within SHA	59
	2. Standards for POCUS equipment support and maintenance standards should be coordinated by each institution within SHA	41

^a^Only potential standards which received at least one vote in support are included. The full set of potential standards is included in the survey in Additional file 1: Appendix S1

The survey results (Table 2) were presented to the 18 attendees at the Roundtable discussion (Table 1). For each domain, informal consensus was sought and obtained on one of the initial proposed standards or a new standard drafted within the session. Several items within the framework generated significant discussion at the Roundtable, likely stemming from the variable stages of development and utilization of POCUS by the multidisciplinary group of stakeholders.

Image capture proved particularly controversial as it related to the domains of documentation in the medical record and quality assurance while the domain of credentials and privileges also required significant discussion to reach consensus. While a standard requiring documentation of POCUS findings in the form of a structured clinical note was widely accepted, there was extensive Roundtable discussion on the capture, storage, and accessibility of POCUS images as they relate to the domains of documentation and quality assurance. Those in favor of image capture saw it as a best practice that should be regularly used at major teaching/training institutions to facilitate indirect supervision of trainee scans as well as quality assurance [29]. Further, it was felt that the ability to share images and/or videos in real time would add value to patient care in cases where a consultant could review pertinent POCUS findings in real time. It was highlighted that in some cases, this may make the difference between a patient staying at a regional site or being transferred to a referral centre. On the other hand, some participants countered that in certain instances image capture seemed an unreasonable requirement.

Rural and regional stakeholders noted that it was not feasible nor cost-effective to implement image capture middleware in every rural centre using POCUS at this time.

It was highlighted that practice audits can be performed by comparing the clinical notes of the POCUS findings with consultative images when these are available. Ultimately, consensus on documentation was reached along with the understanding that the advancement of POCUS technology, and the evolution of POCUS as a distinct imaging modality [21] with its own criteria for image capture, would guide further iterations of the framework.

The consensus built among this group paved the way for broad agreement among the conference attendees. Table 3 outlines the standards endorsed in the plenary session along with the results of the vote totals. Consensus (defined a priori as > 80% agreement) was reached for the standards under each of the domains.

**Table 3:** Consensus domains and plenary session support for each of the standards within the multidisciplinary POCUS framework

Domain	Standard	Support^a^
1. Scope of use	The appropriate application of POCUS should be defined by individual disciplines and be used whenever supported by reasonable evidence	46/48 96%
2. Credentials and privileges	Disciplines should define specific and evolving required credentials that must be met for their providers to receive and maintain privileges to use POCUS. These credentials should be consistent with national standards	49/50 98%
3. Documentation in the medical record	POCUS findings should be documented in the patient chart, much like the physical exam findings as part of the overall clinical assessment. When image capture is available, select POCUS images should be archived and available to support the patient’s ongoing care. When image capture is not available, departments should develop a system to track POCUS to support quality assurance and the patient’s ongoing care	47/49 96%
4. Quality assurance	An audit of POCUS should be coordinated by any groups utilizing POCUS. Review of images, when available, is strongly encouraged. The details of this process should be determined by each discipline	45/49 92%
5. Leadership and governance	Each discipline should oversee its own use of POCUS. A multidisciplinary committee with representatives from each discipline should be formed to collaborate and promote best practices	47/50 94%
6. Teaching	Formal POCUS education and assessment can be provided by those with credentials recognized by their discipline	50/52 96%
7. Research	Clinical and educational research should be encouraged within each department. There should be a concerted effort to coordinate research between disciplines to maximize research productivity to help propel the University of Saskatchewan as a leader in POCUS research	50/53 94%
8. Equipment support and maintenance	Standards for POCUS equipment support and maintenance standards should be coordinated by each discipline as per current best practices and safety guidelines	47/52 90%

^a^Not all conference attendees responded to each of the polls

## Discussion

We have developed a consensus-based multidisciplinary POCUS framework outlining standards for eight domains: scope of use, credentialing and privileges, documentation, quality assurance, leadership and governance, teaching, research, and equipment maintenance. Consensus on modified standards was achieved in the 18 participant Roundtable and endorsed by > 90% of respondents at the conference.

We anticipate that this framework will be used in two ways. Locally, our working group will present our findings to our Health Association’s Provincial Practitioner Advisory Committee as a next step in establishing minimum standards. We expect that these standards will inform the local adoption of discipline-specific guidelines that meet or exceed the requirements we have outlined (our Department of Emergency Medicine has already begun this process). More broadly, we anticipate that our process could be used by health professionals in other jurisdictions to develop their own multidisciplinary framework in a similar consensus-building manner.

Our working group sought to develop a multidisciplinary framework that was both provincial and institutional in application. Foundational to our approach was an understanding that, as per the American Medical Association resolution 802, training and education standards for the use of ultrasound imaging be developed by each physician’s respective specialty [30]. Building from this, our goal was to develop a common framework upon which POCUS will continue to thrive within our institution. Other consensus processes described in the literature have been either specialty specific, hospital-based [26], or part of a broader clinical or billing protocol issued by a provincial College [31]. Notably, the Canadian Association of Radiologists recently published a position statement written exclusively by radiologists with no evidence of input from the POCUS community or any other discipline [32]. We believe our process is more pragmatic and has greater legitimacy in that we utilized a collaborative and consensus-based approach that incorporated the perspectives of multiple disciplines and clinical environments.

One area of discussion which was not anticipated within the pre-developed domains was the question of which instances and images (when captured on a middleware platform) should then be exported to the patient’s permanent medical record. While all agreed that, when possible, scans that significantly impacted medical-decision making should be documented with images and/or video, questions remain regarding the necessity of recording procedural scans (e.g., central venous catheter placement). In addition, it would seem appropriate to consult patients about their preference as well. A subsequent review of the literature on this topic provided limited guidance. Local audits within the department of Emergency Medicine, comparing the written findings with consultative imaging, have revealed a concordance rate in excess of 90% and demonstrated appropriate application and integration of POCUS [33]. As such, there were concerns that the adoption of an image capture requirement would potentially and unjustifiably delay the uptake of POCUS by providers and for patients with the least access to advanced imaging. Further, given the vast range of POCUS applications, several participants suggested that a “one size fits all” approach is likely not appropriate. It was noted that POCUS findings land along a spectrum of clinical meaning and utility, at one end serving as an extension of the physical exam (consider POCUS for jugular venous pressure or interstitial lung syndrome) and the other performing as diagnostic imaging (consider POCUS for intrauterine pregnancy or lower extremity deep venous thrombosis). This spectrum of utility and impact will need to be kept under consideration as image capture standards evolve. As this was not addressed in our current framework, image capture for the patients’ permanent record will need to be determined by both discipline-specific experts as well as our colleagues on the receiving end of transitions in care.

## Limitations

The process of developing the multidisciplinary framework had several limitations. Despite efforts to recruit representatives from all disciplines using POCUS in our province, specialties including Neonatal Critical Care and Physiatry were not involved. Further, although we did have working group representatives from three western Canadian provinces, the majority of our experts were based in one province. Therefore, applicability of our POCUS standards to other jurisdictions will need to be decided on a case-by-case basis. Additionally, we did not grade the strength of each recommendation, as this was not part of our protocol. A significant outstanding question regarding when image capture needs to be used was raised during the Roundtable discussion, but was not addressed through this process. Finally, we believe our multidisciplinary approach would be enriched by having input from colleagues who have traditionally interpreted diagnostic ultrasonography in the fields of cardiology, obstetrics/gynecology, and medical imaging. These specialties were invited to participate in our working group, but unfortunately, there was no response to the invitation.

The adoption of provincial standards will be an iterative process, with members of our working group dedicated to ongoing discussions with our health authority as well as with other colleagues. We plan to reconvene the Roundtable annually at each SASKSONO event to address current standards and evolving issues.

## Conclusion

In conclusion, our multidisciplinary POCUS framework provides a provincial standard upon which each discipline utilizing POCUS can build. It represents one of many initiatives to ensure high-quality use of POCUS that accounts for its use across many different clinical settings (including pre-hospital, the emergency department, the operating room, critical care unit, surgical and medical wards, and outpatient clinics). Locally, it will inform the implementation and utilization of POCUS within the University of Saskatchewan and the Saskatchewan Health Authority. More broadly, this process and its outcomes could be used as a template for the development of multidisciplinary POCUS standards within other jurisdictions.

## Supplementary information

**Additional file 1.** USASK POCUS Framework survey.

**Additional file 2.** Survey results (comprehensive).

## Data Availability

All data generated or analysed during this study are included in this published article and its Additional files.
