# Supplementary material for: The development of a provincial multidisciplinary framework of consensus-based standards for Point of Care Ultrasound at the University of Saskatchewan
Source: Ultrasound J. 2019 Oct 17;11:28. doi: 10.1186/s13089-019-0142-7 (PMC6797680; doi:10.1186/s13089-019-0142-7)
Supplement: Supplementary file 1 — Additional file 1. USASK POCUS Framework survey. [file 13089_2019_142_MOESM1_ESM.docx]

Point of care ultrasound (POCUS) is a diagnostic imaging modality that provides clinically significant data not obtainable by inspection, palpation auscultation, or other components of the physical examination.^[[1]](#endnote-1)^

For each of the categories below, please indicate (enter Yes in the row below) which of the following approaches (you may choose more than 1) you would support:

| **Scope of Use (Application)** | | |
| --- | --- | --- |
| POCUS should only be used in emergencies when conventional medical imaging is not available. | POCUS use should be limited to those applications that cannot be provided by conventional imaging in both emergent and non-emergent situations. | The appropriate application of POCUS should be defined by individual specialties / disciplines and be used whenever supported by reasonable evidence. |
|  |  |  |
| **Credentials/Privileges^[[2]](#endnote-2)^** | | |
| No credentials or additional privileges should be required for the use of POCUS; its use should be up to the clinician similar to any other aspect of the clinical assessment | Any additional training required (and associated privileges) to use POCUS should be determined on a case by case basis by each department | Departments should define specific credentials that are required to receive privileges to use POCUS |
|  |  |  |
| **Documentation in medical record** | | |
| POCUS should be documented in the same way as physical exam findings as part of the overall clinical assessment. | POCUS findings should be documented, and images captured when they play a significant role in patient care decisions. | POCUS findings should be documented, and all images should be captured for inclusion in the patient’s medical record |
|  |  |  |
| **Quality Assurance** | | |
| No specific quality assurance processes need to be in place to use POCUS. | An audit of POCUS should be coordinated by any groups utilizing POCUS. Review of images, when available, is strongly encouraged | POCUS use should include image capture and all images must be reviewed for quality assurance purposes. |
|  |  |  |
| **Leadership & Governance** | | |
| none | Each specialty/discipline oversees its own use | Multidisciplinary committee with representatives from each specialty/discipline using POCUS |
|  |  |  |
| **Teaching** | | |
| POCUS education can be provided by clinicians without specific credentials. | POCUS education can be provided by those with privileges recognized by their Department | POCUS education can be provided only by those with specific credentials as determined by a multidisciplinary POCUS committee. |
|  |  |  |
| **Research** | | |
| None | Clinical and educational/training research should be encouraged within each department | Concerted and coordinated efforts to maximize research productivity to help propel USASK as a leader in POCUS research |
|  |  |  |
| **Equipment Support and Maintenance** | | |
| Standards for POCUS equipment support and maintenance standards should be coordinated by each institution within SHA. | Standards for POCUS equipment support and maintenance standards should be coordinated by each provincial specialty department. | Universal standards for POCUS equipment support and maintenance should be coordinated centrally within SHA . |
|  |  |  |

This document was prepared by a multidisciplinary group of POCUS users from the University of Saskatchewan in collaboration with experts throughout Canada including: Dr. Michelle Clunie (Anesthesia), Dr. Tom Guzowski (Internal Medicine), Dr. Joann Kawchuk (Anesthesia and Critical Care), Dr. Dan Kim (Emergency Medicine), Dr. Peggy Lambos (Pediatrics) Dr. Irene Ma, (Internal Medicine), Dr. Brady Murphy (Family & Emergency Medicine), Dr. Paul Olszynski (Emergency Medicine), Dr. Brent Thoma (Emergency Medicine)

1. American College of Emergency Physicians/ Ann Emerg Med. 2014:64:218 http://dx.doi.org/10.1016/j.annemergmed.2014.05.020 [↑](#endnote-ref-1)
2. Credentials are defined as any documentation demonstrating specific training or skills acquired by that clinician. Privileges are defined as the authorization to a clinician to perform a task(s) by their department (or equivalent) [↑](#endnote-ref-2)
