# Supplementary figures and images for: The development of a provincial multidisciplinary framework of consensus-based standards for Point of Care Ultrasound at the University of Saskatchewan
Source: Ultrasound J. 2019 Oct 17;11:28. doi: 10.1186/s13089-019-0142-7 (PMC6797680; doi:10.1186/s13089-019-0142-7)

Survey Results (shared at SONO-Roundtable, SASKSONO19)


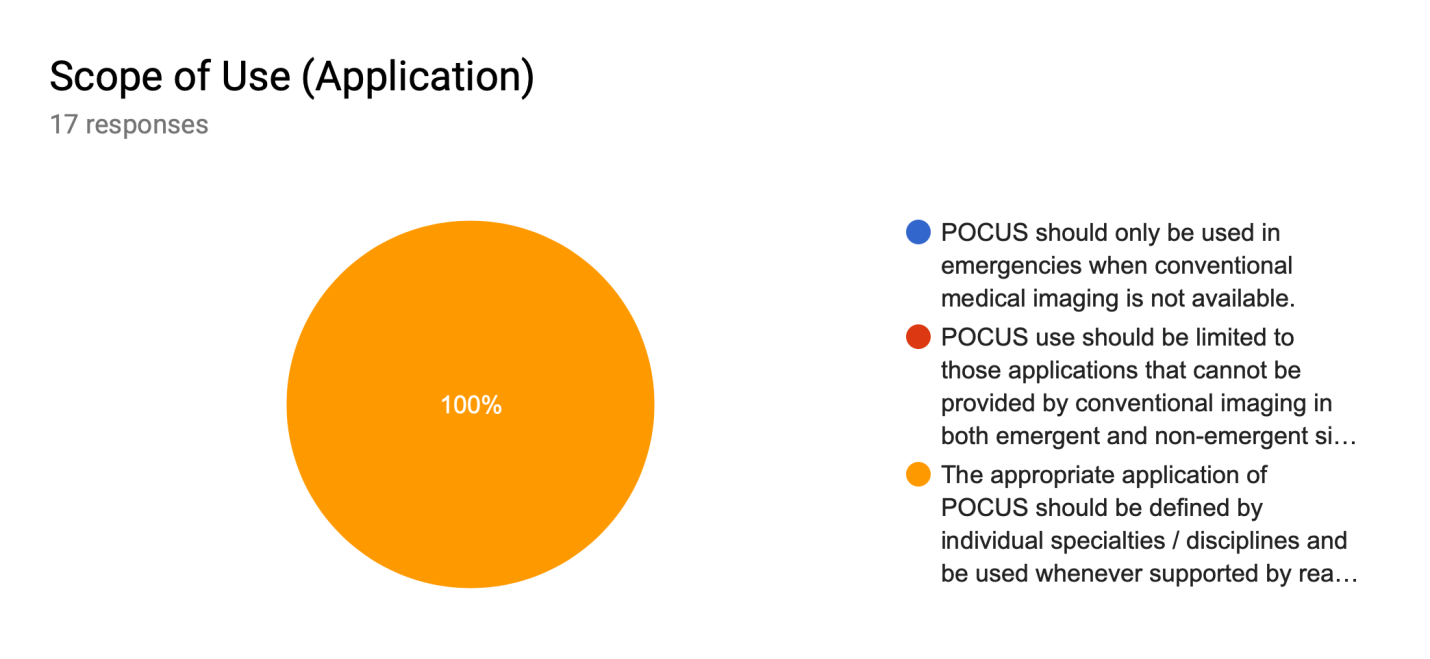


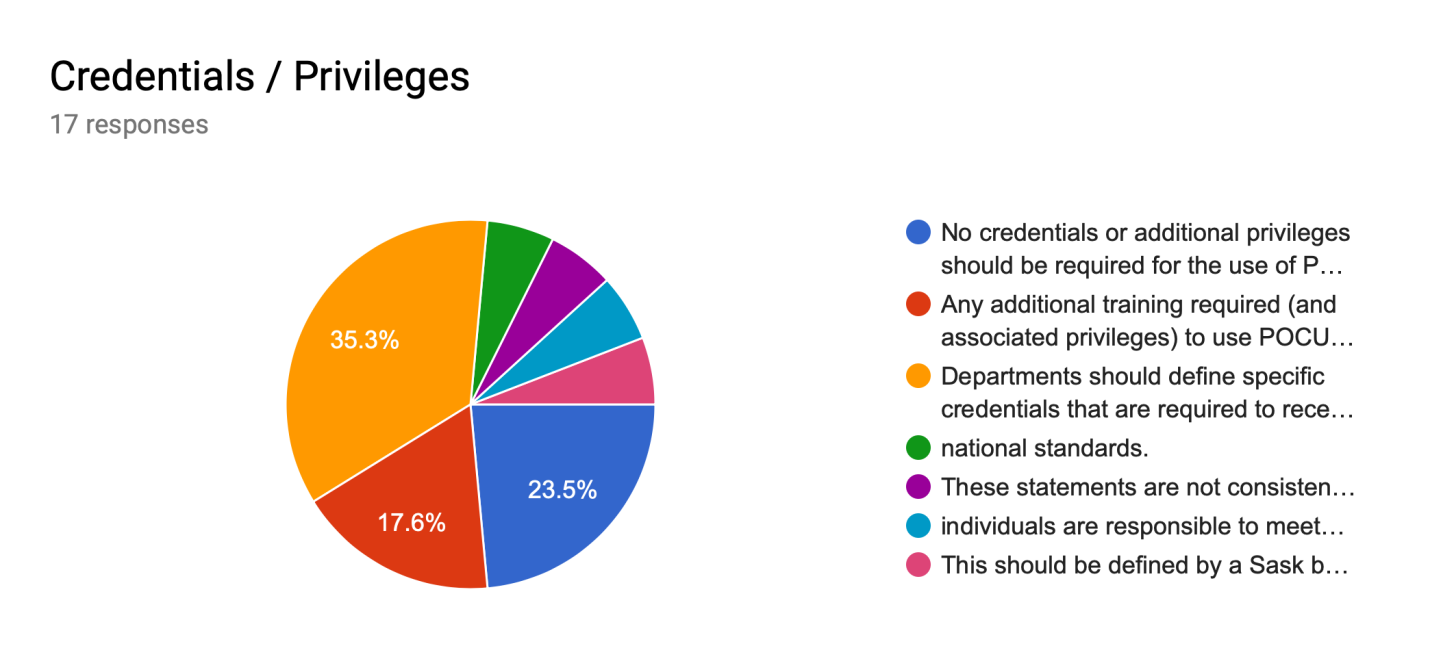


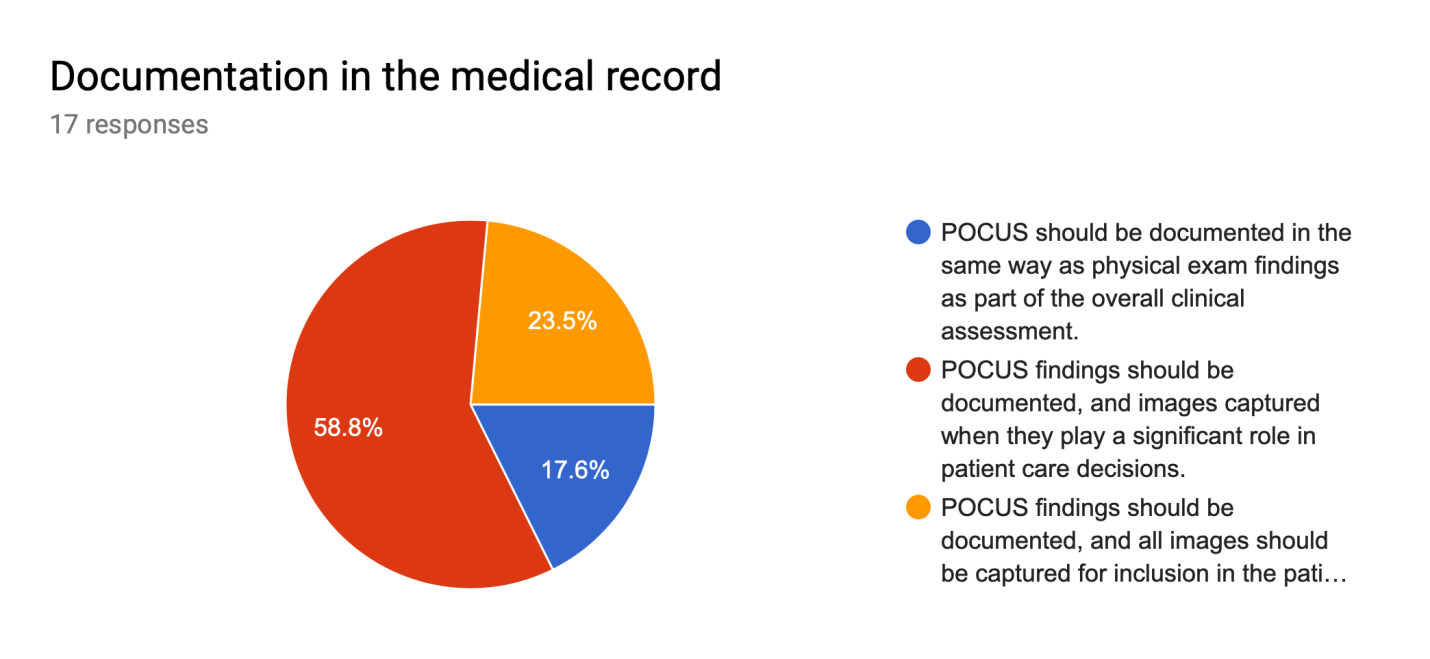


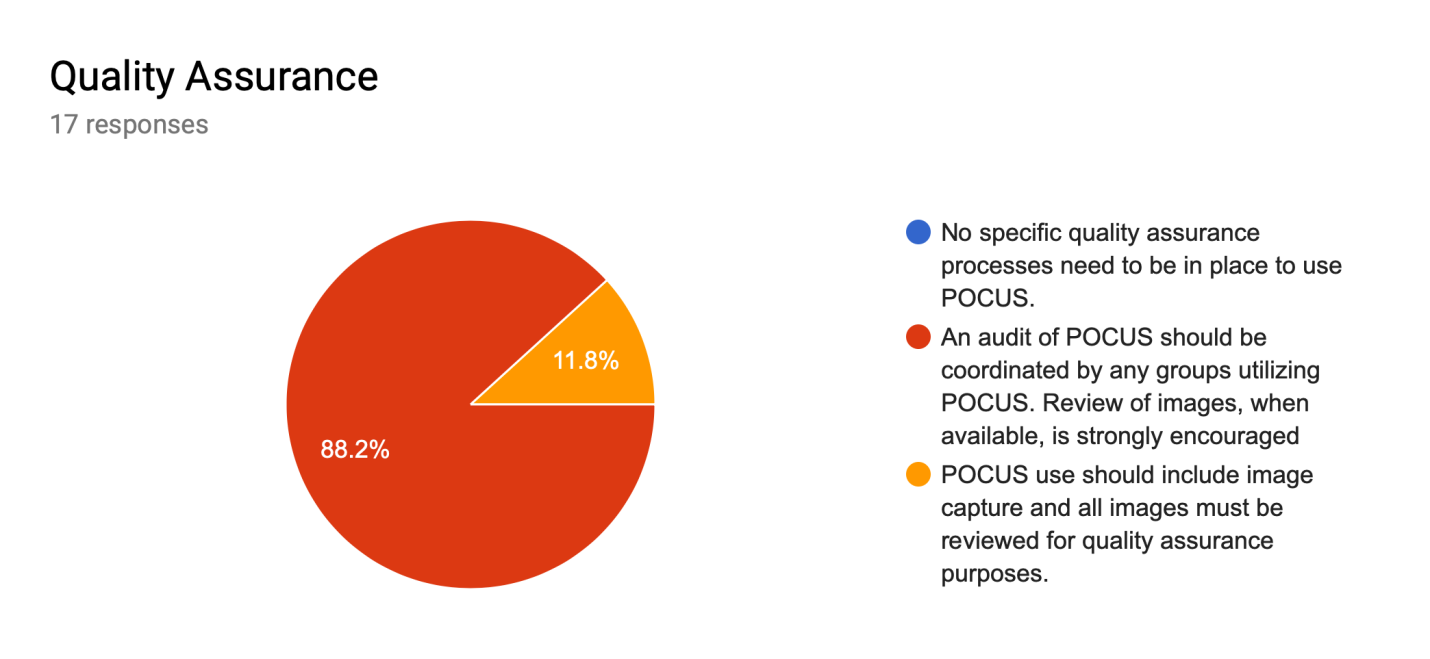


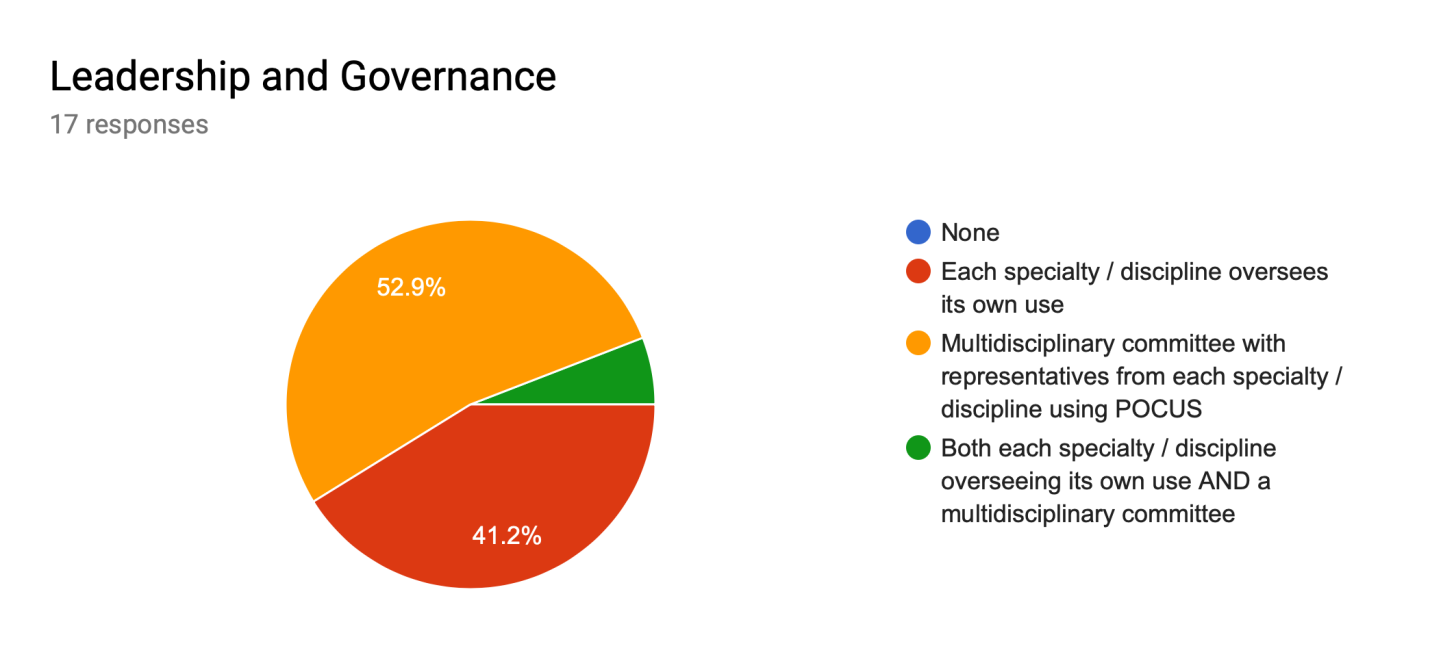


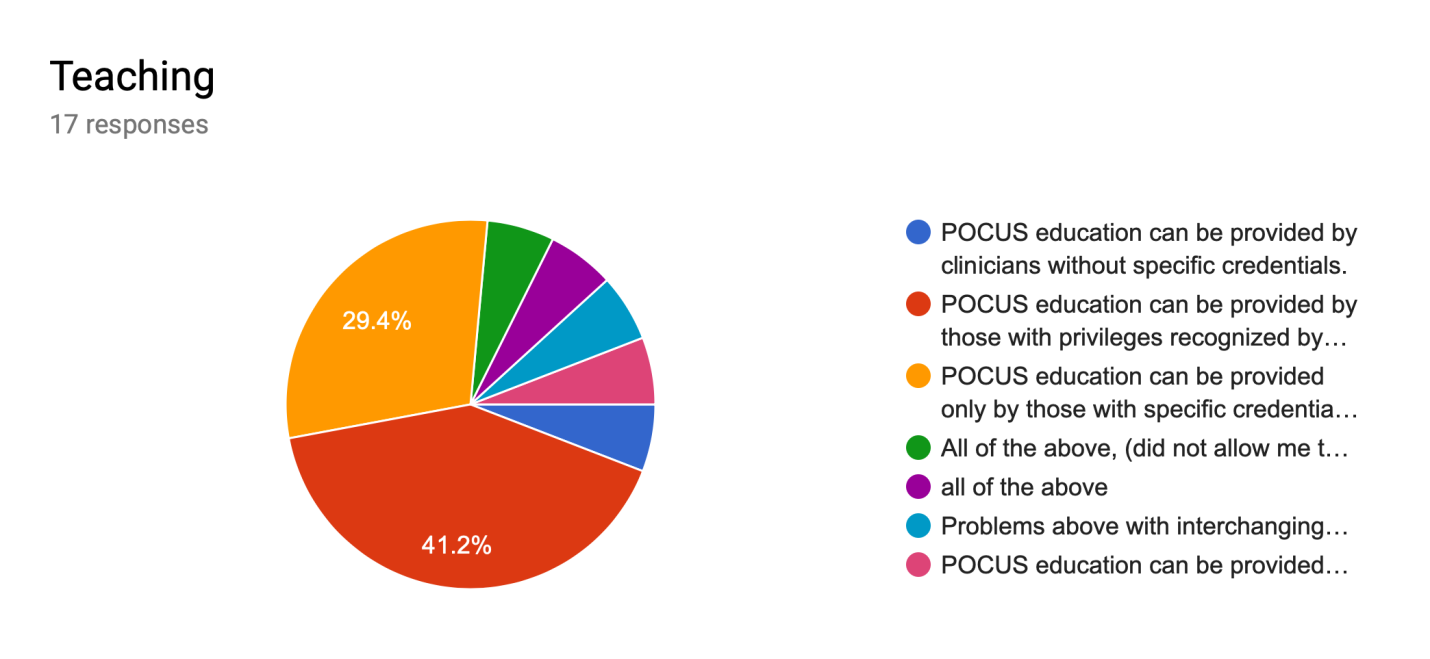


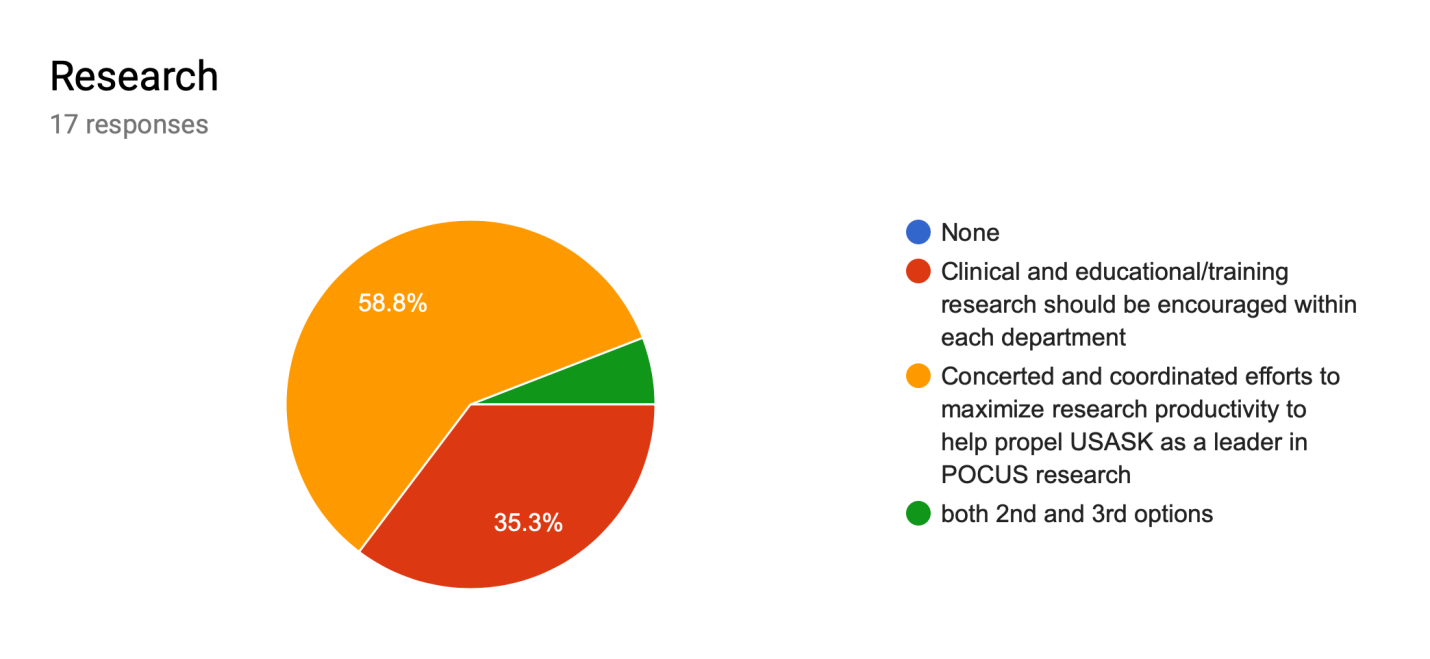


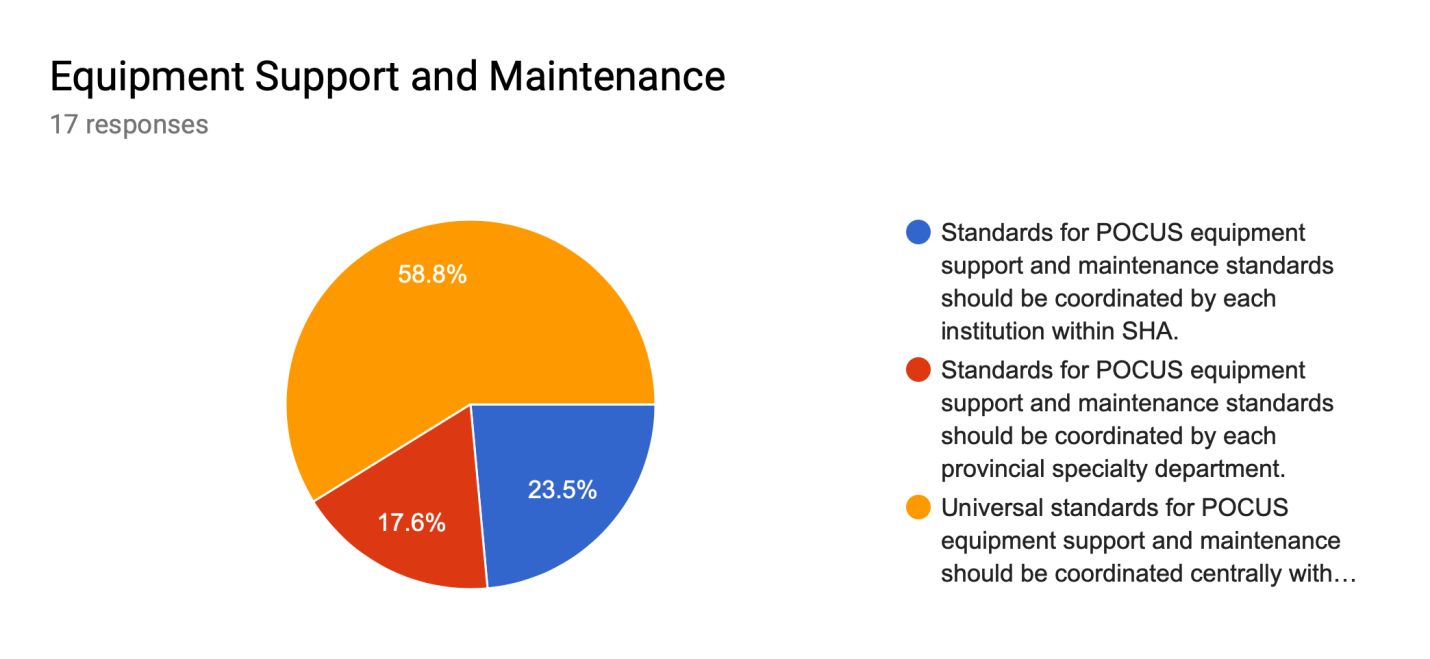

Supplement: Supplementary file 2 — Additional file 2. Survey results (comprehensive). [file 13089_2019_142_MOESM2_ESM.docx]
